# Harmonic analysis of radial pulse in traditional Chinese medicine: physiological alterations associated with hepatitis B and hepatitis C infections

**DOI:** 10.3389/fmed.2026.1848946

**Published:** 2026-07-08

**Authors:** Chung-Hung Wu, Kuan-Fu Liao, Jian-Jung Chen, Hsientsai Wu

**Affiliations:** 1 Department of Chinese Medicine, Taichung Tzu Chi Hospital, Buddhist Tzu Chi Medical Foundation, Taichung, Taiwan; 2 School of Post-Baccalaureate Chinese Medicine, Tzu Chi University, Hualien, Taiwan; 3 Graduate Institute of Acupuncture Science, China Medical University, Taichung, Taiwan; 4 College of Medicine, Tzu Chi University, Hualien, Taiwan; 5 Division of Hepatogastroenterology, Department of Internal Medicine, Taichung Tzu Chi Hospital, Buddhist Tzu Chi Medical Foundation, Taichung, Taiwan; 6 Department of Chinese Medicine, Taichung Tzu Chi Hospital, Buddhist Tzu Chi Medical Foundation, Taichung, Taiwan; 7 Institute of Medical Sciences, Tzu Chi University, Hualien, Taiwan; 8 Department of Electrical Engineering, Dong Hwa University, Hualien, Taiwan

**Keywords:** harmonic analysis, harmonic component, hepatitis B or C virus (HBV or HCV) infection, pulse diagnosis, radial pressure pulse, traditional Chinese medicine

## Abstract

**Background:**

This study investigated whether harmonic analysis (HA) of radial pressure pulse (RPP) signals, a quantitative approach derived from traditional Chinese pulse diagnosis, is associated with physiological alterations in patients with hepatitis B virus (HBV) and hepatitis C virus (HCV) infections.

**Methods:**

A total of 75 participants were enrolled, including 36 healthy controls, 17 patients with HBV infection, and 22 patients with HCV infection. Radial pressure pulse signals were acquired using the Skylark Pulse Analysis System, and harmonic components were calculated from the six radial pulse positions (left and right Cun, Guan, and Chi) following peak alignment and frequency-domain decomposition.

**Results:**

Among the harmonic parameters, the first harmonic component (C1) demonstrated significant differences among the three groups (one-way analysis of variance [ANOVA], *p* = 0.014). *Post hoc* analysis revealed significantly higher C1 values in the HCV group compared with healthy controls (529.09 ± 57.99 vs. 477.78 ± 100.49, *p* = 0.047), whereas the HBV group showed a similar but non-significant trend (503.65 ± 64.51 vs. 477.78 ± 100.49, *p* = 0.481). No significant difference was observed between the HBV and HCV groups (*p* = 0.098). Exploratory logistic regression analysis further demonstrated an independent association between C1 and HCV infection status (OR = 1.008, 95% CI: 1.000–1.017, *p* = 0.047), whereas no significant association was identified for HBV infection.

**Conclusion:**

Alterations in the first harmonic component (C1) of radial pulse waveforms were observed in patients with chronic viral hepatitis, particularly in those with HCV infection. These findings suggest that harmonic analysis may provide complementary information regarding systemic physiological alterations associated with viral hepatitis. However, the exploratory nature of this study, together with the small sample size and modest effect size, warrants cautious interpretation. Larger prospective studies are needed to validate these observations and clarify their clinical significance.

## Graphical abstract

**Figure:**
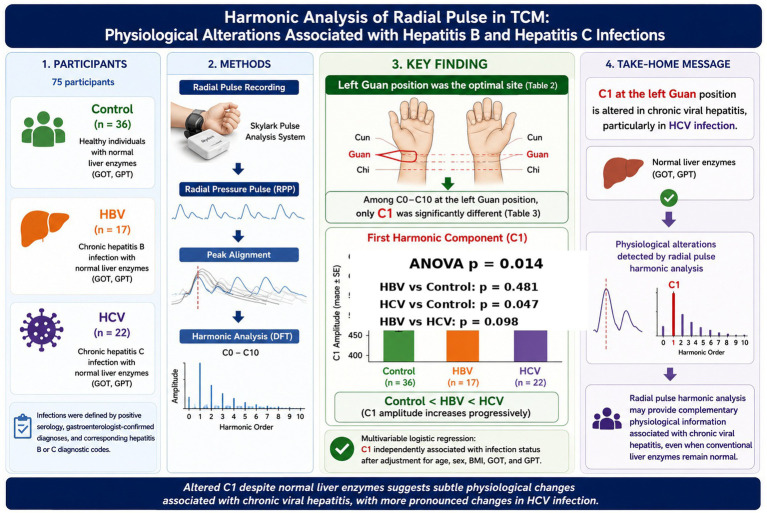

## 1 Introduction

Hepatitis B virus (HBV) and hepatitis C virus (HCV) infections are significant global health issues, affecting millions worldwide and potentially causing severe liver complications (1). In Taiwan, hepatitis B and C viruses are among the most prevalent infectious diseases (2). Early detection is crucial for timely intervention and management. However, current diagnostic methods typically involve invasive blood tests, which may discourage some individuals from seeking screening (3). Traditional Chinese medicine (TCM) has long employed pulse diagnosis as a non-invasive method to evaluate overall health and identify various conditions (4). Recent technological advancements have enabled the quantification and analysis of pulse signals, creating new opportunities for objective diagnosis (5). Several studies have explored the application of TCM principles in hepatitis detection. Chen et al. (2022) investigated anti-HBV constituents from traditional Chinese herbs (6), while Stickel and Schuppan (2007) examined the effects of certain TCM treatments on HBV protein expression (7). However, these studies focused primarily on herbal treatments rather than diagnostic methods. Previous research has demonstrated the potential of pulse diagnosis in identifying liver-related conditions. Li et al. (2022) reported advancements in the non-invasive assessment of non-alcoholic fatty liver disease using pulse characteristics and liver function tests (8). However, there remains a gap in the application of advanced signal processing techniques, such as harmonic analysis (HA), to pulse diagnosis for hepatitis detection.

By combining traditional TCM concepts with modern signal processing techniques, this research seeks to develop a non-invasive, early detection method for HBV/HCV infections. Such an approach could potentially increase screening accessibility and improve early intervention rates. First, the studies by Wang et al. (9, 10) proposed the use of TCM pulse examination and the application of HA in TCM pulse diagnosis. Subsequently, the studies (11, 12) addressed the correlation between harmonic components and specific organ functions. Recently, many scholars have performed HA of RPPs in humans to determine their possible clinical application in TCM. For example, refer to the use of HA for cardiovascular disease detection in the studies mentioned in Refs. (13) and (14). Other researchers have adopted the latter pulse diagnosis instrument to conduct harmonic analyses of the RPPs of hypertension patients (15) and to investigate the role of the RPP in coronary artery disease (16), type 2 diabetes mellitus (T2DM) (17–19), and the preservation of health (20). More importantly, one pulse diagnosis instrument (i.e., the Skylark pulse analysis system) was generally used in the study (21) with incorporating frequency domain indices (e.g., spectral energy [SE] at 0–10, 10–50, and 13–50 Hz), which have been used for further comparison of the results in the study.

According to Wang (10), Chinese medicine assigns fire, wood, water, earth, and metal (i.e., the five fundamental element types in the material world) could be mapped to the heart (harmonic zero, C0), liver (first harmonic, C1), kidney (second harmonic, C2), spleen (third harmonic, C3), and lungs (fourth harmonic, C4), respectively. The values of C0–C4 are the first five representative coefficients of the harmonic component of the RPP in HA. More importantly, there have been further studies on C0–C4 within the field of HA—for example, as a prognostic indicator of a protective factor in the prediction of type 2 diabetes using C0 (22), and as a favorable predictive indicator in the monitoring of coronary artery disease using the harmonic C1 (16). C1 has also been regarded as an independent predictor for major adverse cardiovascular events, cardiovascular mortality, and microvascular events, as discussed elsewhere (13, 19). It is a powerful independent predictor of most cardiovascular complications in patients with T2DM alongside C4 (17). Therefore, improved software for digitalizing the RPP and computing coefficients of the harmonic components for HA has been developed in response to the urgent need to increase the efficacy of pulse diagnosis in TCM. It is reasonable to infer that variations in the average weight of a specific harmonic reflect both physical and mental characteristics; however, more evidence is needed from additional studies. To date, only a small number of studies have investigated HA of radial pulse waveforms in patients with HBV or HCV infections.

The present study explored whether harmonic characteristics derived from radial pressure pulse (RPP) signals are associated with physiological alterations in individuals with chronic viral hepatitis. Using the Skylark pulse analysis system, 6-s RPP recordings were obtained from six pulse positions. Harmonic components were subsequently calculated using a modified HA approach incorporating peak alignment. A total of 75 participants were enrolled, including 36 healthy controls, 17 patients with HBV infection, and 22 patients with HCV infection, all of whom had serum glutamic oxaloacetic transaminase (GOT) and glutamic pyruvic transaminase (GPT) levels within the normal reference ranges. The objectives of this study were twofold. First, we evaluated the feasibility of applying HA to RPP signals acquired using the Skylark pulse analysis system. Second, we investigated whether harmonic parameters, particularly the first harmonic component (C1), were associated with HBV and HCV infection status and could provide insight into the physiological alterations associated with chronic viral hepatitis.

## 2 Materials and methods

### 2.1 Subject grouping and study protocol

#### 2.1.1 Study protocol

This study employed a pulse diagnosis instrument (the Skylark pulse analysis system) to acquire 6-s RPP recordings from six pulse positions, including the Cun, Guan, and Chi positions on both wrists. In addition, the mean values of the spectral energy indices (SE0–10, SE10–50, and SE13–50 Hz) were calculated. Liver enzyme parameters and RPP measurements were obtained on the same day.

The study aimed to evaluate harmonic characteristics derived from RPP signals using a modified HA approach and to explore their associations with chronic viral hepatitis. Comparisons were performed among healthy control subjects, patients with HBV infection, and patients with HCV infection to investigate whether specific harmonic components, particularly the C1, were associated with physiological alterations related to viral hepatitis.

#### 2.1.2 Grouping and clinic visits for hepatitis

Anthropometric, demographic, pulse diagnosis, and medical history data were collected during routine clinical visits over a six-month recruitment period. For each participant, all measurements were obtained during a single study visit. Blood samples were collected on the same day as pulse waveform acquisition. In selected cases, abdominal ultrasonography was performed at the attending physician’s discretion.

A total of 83 participants underwent pulse waveform assessment using the Skylark pulse analysis system. Patients with chronic viral hepatitis were recruited from the hepatitis outpatient clinic. Eight participants were excluded because of a history of hepatitis-related complications, including liver cirrhosis or hepatocellular carcinoma. Consequently, 75 participants were included in the final analysis.

The study population consisted of three groups: healthy controls (*n* = 36), patients with HBV infection (*n* = 17), and patients with HCV infection (*n* = 22). Healthy controls tested negative for both HBV and HCV infection. HBV and HCV infections were identified based on positive serological findings, gastroenterologist-confirmed clinical diagnoses, and corresponding hepatitis B or hepatitis C diagnostic codes recorded in hospital medical records. To minimize potential measurement bias, group allocation was concealed during pulse waveform acquisition and HA. All participants underwent identical measurement procedures, and both the technicians responsible for data acquisition and the investigators performing data analysis were blinded to group assignment during measurement and analysis.

#### 2.1.3 Data collection

The subjects were taken to the outpatient clinic at Taichung Tzu Chi Hospital for medical assessment and blood sampling, including determination of GOT and GPT. The subjects waited outside the clinic until the doctor’s assessment was complete. After the doctor’s assessment was completed, the subjects were taken to a health clinic to record physiological data (i.e., age, body weight, and height) and to complete the questionnaire. Subsequently, the subjects were asked to take a 10 min rest. All subjects were allowed to assume a sitting position in a relaxed manner in a quiet, humidity- and temperature-controlled room at 26 ± 1 °C for RPP measurements. For each localized measurement (the Cun, Guan, and Chi positions on the left and right wrists), RPP waveforms were recorded for a duration of 6 s. The frequency domain indices (i.e., spectral energies [SEs] at 0–10, 10–50, and 13–50 Hz) were obtained using the Skylark pulse analysis system.

### 2.2 Description of the pulse diagnosis instrument and data acquisition

#### 2.2.1 Skylark pulse analysis system

The Skylark pulse analysis system (Taiwan Food and Drug Administration [TFDA] medical equipment license No.: 002302) consists of two parts: (1) an X–Y–Z axial moveable frame connected to a pressure sensor with a precision of ±5 mm Hg and an operating range of 80–120 mm Hg, which can record 6 s of arterial pulsation at a sampling rate of 3,000 Hz at each location; (2) a computing unit with software for digitalizing the RPP and producing frequency domain indices. The pressure sensor operated within a range of 80–120 mm Hg, corresponding to the depth in traditional Chinese pulse diagnosis (21). To ensure consistency, the system automatically recorded the RPP signals at their peak amplitude. This process, overseen by a single technician, guaranteed that measurements were taken at equivalent depths across all subjects. The RPP values at the left Cun, left Guan, left Chi, right Cun, right Guan, and right Chi locations for each subject were recorded over 10 min by a single technician trained to operate the system. The system automatically recorded the RPP signals and several indices (i.e., spectral energies (SEs) at 0–10, 10–50, and 13–50 Hz) at six locations in Microsoft Excel format. The system analyzed the raw RPP data from the six locations to develop several novel quantitative indices for pulse diagnosis.

#### 2.2.2 Radial pressure pulse signal acquisition using the skylark pulse analysis system

**Figure 1:**
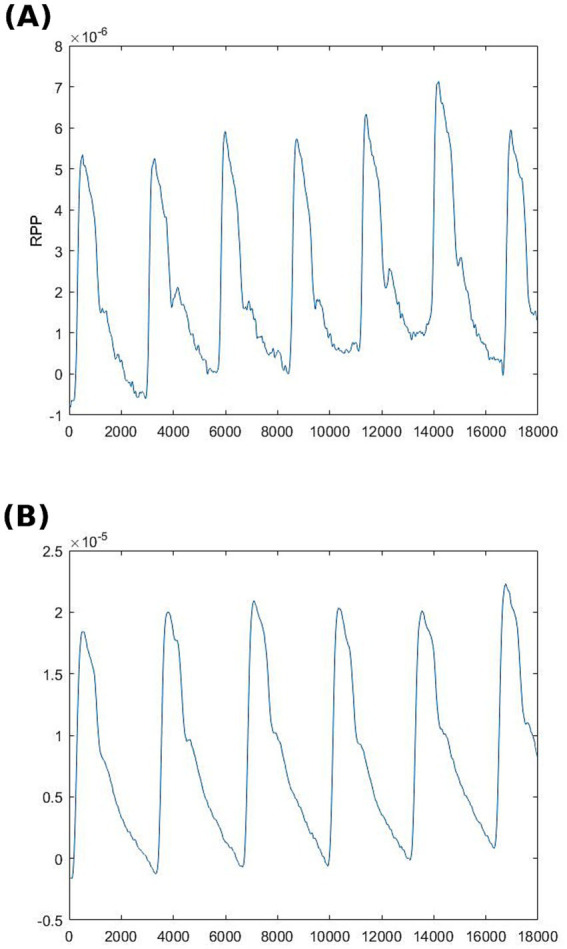
The pulse signals were acquired from the Skylark pulse analysis system. Radial pressure pulse (RPP) signal acquisition from the Guan position on the left wrist **(A)** for subject A (healthy older individual aged 73 years; GOT = 24, GPT = 27) with regular multivitamin intake and **(B)** for subject B (hepatitis C virus (HCV)-infected patient aged 64 years; GOT = 34, GPT = 38) without regular multivitamin intake. Both waveforms consisted of 18,000 consecutive data points of data sampled over 6 s at a sampling frequency of 3,000 Hz sampling frequency. The radial pulse signals of 18,000 samples are shown.

Similar to study mentioned in Ref. (23), the Cun, Guan, and Chi pulse positions on both wrists were examined with the patient in a natural sitting posture, with the forearm level with the heart and the elbow bent to and angle of 120°. The RPP was recorded when the Skylark pulse analysis system displayed the greatest amplitude. The subjects were not allowed to speak during the measurements. Conventionally, TCM physicians palpate the RPP at three adjacent regions of the radial artery on the left and right wrists, namely, the Cun (distal), Guan (middle), and Chi (proximal) pulses. Each pulse position corresponds to a specific visceral organ in the human body, as shown in Ref. (24). After the pulse position is located, a pressure sensor must be placed at the radial artery pulse to obtain optimal RPP waveforms using the Skylark pulse analysis system. Graphical and digital presentations of the SE at 0–10 Hz (SE0–10 Hz), 10–50 Hz (SE10–50 Hz), and 13–50 Hz (SE13–50 Hz) were provided instantly by the software package during the assessments. As shown in Figure 1, using an automatically digitalized representation using an analog-to-digital (A/D) converter, the RPP waveforms could be represented by a fundamental frequency sine wave and a collection of harmonics of that fundamental sine wave, and summed together linearly (i.e., discrete time Fourier series) in the HA application (16). Subsequently, the newly developed HA method could be applied to the RPPs as described in Section 2.3.

### 2.3 Harmonic analysis (HA) of the radial pressure pulse

The HA of RPP in the current study comprised two processes: (1) the peak alignment process and (2) the harmonic component’s computation.

#### 2.3.1 Peak alignment process

RPPs were adopted for computing harmonic components after the peak alignment process. In this study, we retrieved consecutive 6-s RPPs according to the locations of the peaks of the pulses, shown as {y[*n*]} = {y[1], y[2], y[3], …, y[*N*]} (for RPP data with *N* samples), with five to *n*p different periods, that is, T_1_, T_2_, …, T_*n*p_. If we assume that T_1_ = T_2_ = … = T_*n*p_ = 1 s, then *N* = 18,000 at a sampling rate of 3,000 Hz. In this case, {y[*n*]} is a sampled period’s discrete signal, and thus, {y[*n*]} = {y[*n* + k × 3,000]}, k = 0, 1, 2, …, 7. These sampled data can be decomposed as follows:


$$\{y[n]\}=\frac{1}{3,000}\sum_{k=0}^{3,000-1}Y(k)e^{i\frac{2\pi }{3000}kn},n=1,2,\ldots ,18,000,$$ (1)

where {**Y**(k), k = 0, 1, 2, …, 10} is the first 11 harmonic components of y[*n*], as suggested in Ref. (25). It can also be represented as follows:


$$\{Y(k)\}=\sum_{n=0}^{3000-1}y[n]e^{-i\frac{2\pi }{3000}kn},k=0,1,2,\ldots ,10.$$ (2)

**Figure 2:**
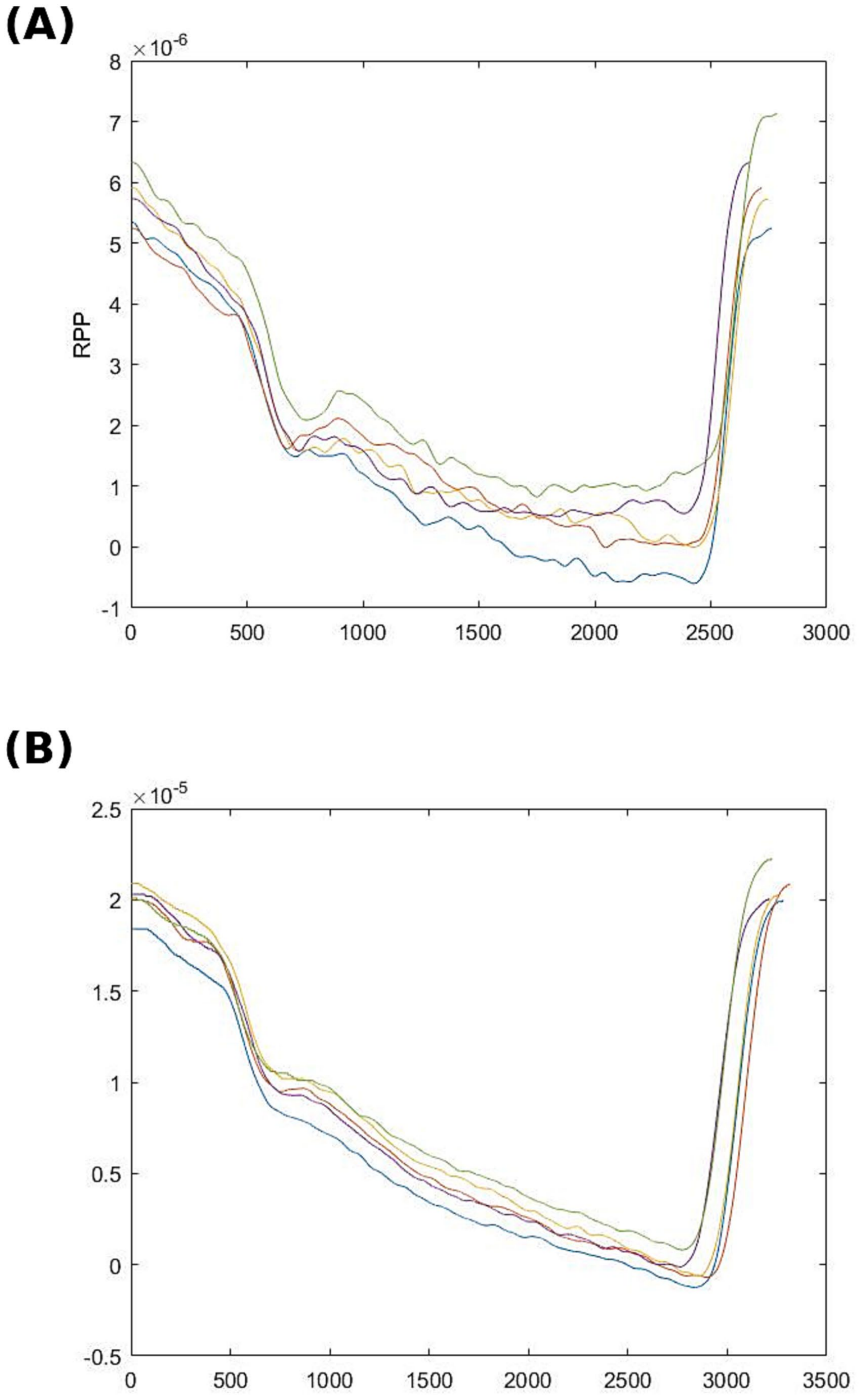
Harmonic analysis of the radial pressure pulse with peak alignment process. Peak alignment for 6-s RPPs. **(A)** Subject A (a healthy older individual aged 73 years; GOT = 24, GPT = 27); and **(B)** Ssubject B (an HCV-infected patient aged 64 years; GOT = 34, GPT = 38), the same subjects as in Figure 1. The 6-s RPP is a quasiperiodic signal; thus, T1 ≈ T2 ≈ … ≈ Tnp are similar but not identical.

Due to the RPP being a quasiperiodic signal, all values of T_i_ (T_1_, T_2_, …, T_*n*p_) are similar, but not identical, in Equations 1, 2. All RPP signals were sequentially separated via peak alignment process. Thus, the total np periods of the RPP could be determined precisely using the ensemble averaging process after peak localization, as shown in Figure 2.

#### 2.3.2 Harmonic component computation

All RPP signals were sequentially separated by the np peaks after the peak alignment process. In addition, the computation of the harmonic component proceeded as follows:

Step 1. The Fourier series coefficients for each period of the RPP were found in Equation 3:


$$\{Y(k,j)\}=\sum_{n=0}^{3,000-1}y_{j}[n]e^{-i\frac{2\pi }{3,000}\mathrm{kn}},k=0,1,2,\ldots ,10,$$ (3)

where y_j_[*n*] indicates the *j*th-period pulse and k indicates the *k*th harmonic component of the *n*p period of the RPP.

Step 2. The averaging amplitude value of each period of the RPP was calculated:


$$|Y(k,j)|=\frac{1}{3,000}\sum_{j}|y_{j}[n]|,j=1,2,\ldots ,np.$$ (4)

The mean of the j vectors of {**Y**(k, j)} is a vector with j means in Equation 4.

Step 3. The coefficients of the Fourier series for each period were normalized into the format {Y(k,j)}/∣Y(k,j)∣ , j = 1, 2, 3, …, *n*p. The *k*th row of period j of {**Y**(k, j)} was divided by the absolute mean of {**Y**(k, j)} of the *j*th period.

Step 4. The representative coefficients of the harmonic component were found as follows:


$$C_{k}=\frac{1}{np}\sum_{j}\frac{\left\{Y(k,j)\right\}}{|Y(k,j)|},k=0,1,2,\ldots ,10;j=1,2,\ldots ,np.$$ (5)

C_0_ in Equation 5 is defined as the averaged total pulse energy of the averaged pulse waveforms for the RPP signals. The other coefficients of the harmonic component (i.e., C_1_–C_10_ in Equation 5) reflect the harmonic components between the heart rate and the harmonic frequencies of the arterial system (18, 19). Finally, as listed in Step 4, the normalized Fourier coefficient was determined as the mean of the normalized harmonic components calculated over the RPP periods included in Equation 5.

### 2.4 Statistical methods

Statistical analyses were performed using the Statistical Package for the Social Sciences (SPSS), version 14.0 (SPSS Inc., Chicago, IL, USA). Continuous variables are presented as mean ± standard deviation (SD). Data normality was evaluated using the one-sample Kolmogorov–Smirnov test. Depending on the data distribution, between-group comparisons were performed using independent-sample *t*-tests, one-way analysis of variance (ANOVA) with Dunnett *post hoc* testing, or the Mann–Whitney–Wilcoxon test, as appropriate. A two-sided *p*-value < 0.05 was considered statistically significant. To investigate factors associated with viral hepatitis status, logistic regression analyses were performed. Initially, univariate logistic regression analyses were conducted for demographic variables (age, sex, BMI), clinical parameters (GOT and GPT), and harmonic components (C_0_–C_10_). Variables that showed a potential association with infection status in the univariate analyses (*p* < 0.25) were included in the multivariable logistic regression models. Backward stepwise elimination was subsequently performed to obtain a parsimonious model, and variables with *p* < 0.05 were retained in the final model. This approach was used to identify independent variables associated with viral hepatitis status while minimizing model overfitting.

Three exploratory logistic regression models were constructed: (1) combined viral hepatitis (HBV + HCV) vs. healthy controls, (2) HBV infection vs. healthy controls, and (3) HCV infection vs. healthy controls. Multicollinearity was assessed using variance inflation factors (VIFs), with VIF > 5 considered indicative of problematic collinearity. Model calibration was evaluated using the Hosmer–Lemeshow goodness-of-fit test, and classification performance was assessed using the overall percentage of correctly classified cases. To further evaluate the potential influence of sex on infection status, an additional logistic regression analysis using sex as the sole predictor variable was also performed.

## 3 Results

In this study, RPP signals and frequency-domain indices were acquired using the Skylark pulse analysis system. Following peak alignment, the RPP waveforms were decomposed into the harmonic zero component (C_0_) and the first 10 harmonic components (C_1_–C_10_) using HA. A total of 75 participants were included in the final analysis, comprising 36 healthy controls, 17 patients with HBV infection, and 22 patients with HCV infection. The analyses were performed in three stages. First, demographic characteristics and liver enzyme parameters were compared among the three study groups (Section 3.1). Second, harmonic characteristics of the RPP signals obtained from the Cun, Guan, and Chi positions were evaluated to identify harmonic components associated with viral hepatitis status (Sections 3.2 and 3.3). Finally, exploratory logistic regression analyses were conducted to assess the association between C_1_ and viral hepatitis status (Section 3.4).

### 3.1 Demographic characteristics and liver enzyme parameters

The demographic characteristics and liver enzyme parameters of the study participants are summarized in Table 1. The study population consisted of healthy controls (*n* = 36), patients with HBV infection (*n* = 17), and patients with HCV infection (*n* = 22). No statistically significant differences were observed among the three groups with respect to age, body height, body weight, body mass index (BMI), GOT, or GPT levels (all *p* > 0.05). Similarly, sex distribution did not differ significantly among groups (*χ*^2^ test, *p* = 0.140). Importantly, liver enzyme parameters remained within the normal clinical range and did not differ significantly among the three groups, indicating that the HBV and HCV participants included in this study generally exhibited preserved liver function at the time of assessment.

**Table 1:** Demographic characteristics and liver enzyme parameters of healthy controls, patients with hepatitis B virus (HBV) infection, and patients with hepatitis C virus (HCV) infection.

Parameter	Control	HBV	HCV	ANOVA *p-*value
	*n* (%) or Mean ± SD	*n* (%) or Mean ± SD	*n* (%) or Mean ± SD	
Sex (male/female)	36 (8/28)	17 (7/10)	22 (10/12)	0.140
Age, year	53.17 ± 17.28	62.71 ± 9.05	57.68 ± 11.98	0.078
Body height, cm	160.22 ± 7.92	163.06 ± 8.75	159.68 ± 7.71	0.384
Body weight, kg	58.33 ± 13.14	61.41 ± 10.32	60.32 ± 10.32	0.639
BMI, kg/m^2^	22.62 ± 4.03	23.09 ± 3.43	23.63 ± 3.65	0.617
GOT, U/L	18.94 ± 5.24	21.00 ± 5.37	20.23 ± 6.16	0.416
GPT, U/L	22.56 ± 7.65	27.35 ± 7.64	24.91 ± 6.91	0.090

Values are presented as *n* (%) or mean ± SD. Continuous variables were compared using one-way ANOVA, and categorical variables were compared using the *χ*^2^ test, as appropriate.

### 3.2 C_1_ for pulse diagnosis at the cun, guan, and chi positions on the left and right wrists

No significant differences were observed among the control, HBV, and HCV groups for any of the frequency-domain indices of the RPP, including SE0–10 Hz, SE10–50 Hz, and SE13–50 Hz, measured at the Cun, Guan, and Chi positions on both wrists (all *p* > 0.05).

Table 2 summarizes the C_1_ of the RPP signals measured at the Cun, Guan, and Chi positions on both wrists in healthy controls, patients with HBV infection, and patients with HCV infection.

**Table 2:** The first harmonic component C1 of harmonic analysis (HA) for the RPP signals from Control Group (asymptomatic subjects who tested negative for both HBV and HCV), HBV Group (patients with only HBV infection), and HCV Group (patients with only HCV infection) at the Cun, Guan, and Chi positions on the left and right wrists.

Signal position	Control	HBV	HCV	ANOVA *p-*value
	*n* = 36	*n* = 17	*n* = 22	
Left Cun position	478.42 ± 97.18	465.94 ± 84.04	507.00 ± 73.54	0.312
Left Guan position	477.78 ± 100.49	503.65 ± 64.51	529.09 ± 57.99*	**0.014**
Left Chi position	472.44 ± 98.61	447.47 ± 76.24	486.59 ± 85.84	0.408
Right Cun position	441.25 ± 85.15	464.71 ± 52.15	481.73 ± 65.47	0.123
Right Guan position	458.33 ± 88.22	495.53 ± 87.14	487.45 ± 92.26	0.281
Right Chi position	463.25 ± 93.01	494.41 ± 77.91	486.73 ± 95.75	0.430

Values are presented as mean ± standard deviation (SD). Differences among healthy controls, patients with hepatitis B virus (HBV) infection, and patients with hepatitis C virus (HCV) infection were evaluated using one-way analysis of variance (ANOVA). When a significant overall group effect was identified, post hoc comparisons were performed using Dunnett’s two-sided test with the control group as the reference. *p < 0.05 vs. the control group (HCV vs. Control).

Among the six pulse positions examined, a significant difference in C_1_ was observed only at the Guan position on the left wrist (one-way ANOVA, *p* = 0.014). No significant differences were identified at the remaining pulse positions, including the left Cun, left Chi, right Cun, right Guan, and right Chi positions (all *p* > 0.05).

At the left Guan position, the mean C_1_ values were 477.78 ± 100.49 in the control group, 503.65 ± 64.51 in the HBV group, and 529.09 ± 57.99 in the HCV group. The assumption of homogeneity of variance was satisfied (Levene’s test, *p* = 0.076). *Post hoc* Dunnett analyses revealed that the HCV group exhibited significantly higher C_1_ values than the control group (*p* = 0.047). In contrast, although the HBV group also showed higher mean C_1_ values than the control group, the difference was not reach statistically significant (*p* = 0.481). No significant difference was observed between the HBV and HCV groups (*p* = 0.098).

Overall, C_1_ values at the left Guan position demonstrated an increasing trend from healthy controls to HBV and HCV patients. These findings suggest that the left Guan position may be the most informative pulse location for detecting harmonic alterations associated with chronic viral hepatitis, particularly HCV infection.

### 3.3 Harmonic components at the left guan position

Table 3 summarizes the harmonic zero component (C_0_) and the first 10 harmonic components (C_1_–C_10_) derived from RPP signals measured at the Guan position on the left wrist in healthy controls, patients with HBV infection, and patients with HCV infection. Among all harmonic components examined, only the C_1_ demonstrated a statistically significant difference among the three groups (one-way ANOVA, *p* = 0.014). The mean C_1_ values increased progressively from the control group (477.78 ± 100.49) to the HBV group (503.65 ± 64.51) and the HCV group (529.09 ± 57.99). *Post hoc* Dunnett analysis revealed that the HCV group exhibited significantly higher C_1_ values than the control group (*p* = 0.047), whereas the HBV group showed a similar but non-significant increase (*p* = 0.481).

**Table 3:** Harmonic components (C0–C10) of radial pressure pulse signals at the Guan position on the left wrist in healthy controls, patients with hepatitis B virus (HBV) infection, and patients with hepatitis C virus (HCV) infection.

Coefficient	Control	HBV	HCV	ANOVA *p-*value
	*n* = 36	*n* = 17	*n* = 22	
C_0_	1,130.47 ± 159.05	1,106.29 ± 204.60	1,088.86 ± 159.34	0.656
C_1_	477.78 ± 100.49	503.65 ± 64.51	529.09 ± 57.99*	**0.014**
C_2_	240.92 ± 50.73	235.06 ± 52.93	235.91 ± 47.58	0.898
C_3_	142.94 ± 42.72	136.71 ± 34.26	138.68 ± 29.43	0.828
C_4_	77.64 ± 24.93	71.18 ± 10.49	81.86 ± 22.68	0.321
C_5_	57.81 ± 19.19	61.88 ± 15.46	63.50 ± 16.33	0.457
C_6_	41.94 ± 14.85	46.53 ± 10.28	49.64 ± 19.47	0.221
C_7_	26.11 ± 9.07	27.00 ± 9.07	32.18 ± 11.47	0.081
C_8_	20.00 ± 10.10	18.29 ± 6.24	20.77 ± 7.20	0.663
C_9_	15.11 ± 7.26	14.65 ± 5.29	16.00 ± 6.27	0.802
C_10_	13.14 ± 9.14	10.94 ± 4.56	12.36 ± 3.95	0.573

Values are presented as mean ± standard deviation (SD). HBV, hepatitis B virus; HCV, hepatitis C virus. Differences among groups were evaluated using one-way analysis of variance (ANOVA). When a significant overall group effect was identified, post hoc comparisons were performed using Dunnett’s two-sided test with the control group as the reference. *p < 0.05 vs. the control group (HCV vs. Control).

No significant differences were observed for the remaining harmonic components (C_0_ and C_2_–C_10_) among the three groups (all *p* > 0.05). Although the seventh harmonic component (C_7_) showed a tendency toward group differences (*p* = 0.081), this trend did not reach statistical significance.

Overall, these findings suggest that alterations in C_1_ may reflect physiological changes associated with chronic viral hepatitis, particularly in patients with HCV infection.

### 3.4 Exploratory logistic regression analysis

Table 4 summarizes the multivariable logistic regression analyses performed to evaluate the association between harmonic parameters and viral hepatitis status. In the combined viral hepatitis model (HBV + HCV vs. healthy controls), C_1_ remained independently associated with infection status after adjustment for sex. Specifically, higher C_1_ values were associated with an increased likelihood of belonging to the viral hepatitis group (OR = 1.008, 95% confidence interval [CI]: 1.002–1.015, *p* = 0.038). Sex was also independently associated with viral hepatitis status (*p* = 0.015). The Hosmer–Lemeshow goodness-of-fit test indicated acceptable model calibration (*p* = 0.388).

**Table 4:** Multivariable logistic regression analyses for combined viral hepatitis, HBV, and HCV infection status.

Model	Comparison	*n*	Sign. HA parameter, *p*-value	OR of C_1_	95% CI	*p*-value of Hosmer–Lemeshow test
#1	(HBV + HCV) vs. Control	75	C_1_, *p* = 0.038 Sex, *p* = 0.015	1.008	1.002–1.015	0.388
#2	HBV vs. Control	53	C_1_, *p* = 0.481	1.001	0.992–1.010	0.248
#3	HCV vs. Control	58	C_1_, *p* = 0.047	1.008	1.000–1.017	0.196

Odds ratios (ORs) and 95% confidence intervals (CIs) were estimated using multivariable logistic regression analyses. Model 1 compared participants with chronic viral hepatitis (HBV + HCV) with healthy controls; Model 2 compared patients with HBV infection with healthy controls; and Model 3 compared patients with HCV infection with healthy controls. Candidate variables (*p* < 0.25 in univariate analyses) were entered into the multivariable models and retained using a backward stepwise selection procedure. Model calibration was assessed using the Hosmer–Lemeshow goodness-of-fit test. The HBV and HCV subgroup analyses should be considered exploratory given the relatively small sample sizes. HBV, hepatitis B virus; HCV, hepatitis C virus; OR, odds ratio; CI, confidence interval.

To further address the distinct clinical characteristics of HBV and HCV infections, separate subgroup analyses were subsequently performed. In the HBV model, C_1_ was not significantly associated with HBV infection status (OR = 1.001, 95% CI: 0.992–1.010, *p* = 0.481). In contrast, the HCV model showed a significant association between C_1_ and HCV infection status (OR = 1.008, 95% CI: 1.000–1.017, *p* = 0.047). The corresponding Hosmer–Lemeshow test results suggested acceptable calibration for both subgroup models (HBV: *p* = 0.248; HCV: *p* = 0.196).

Overall, the significant association observed in the combined viral hepatitis model was primarily driven by the HCV subgroup, whereas the HBV subgroup showed a similar trend but did not reach statistical significance. These findings are consistent with the group comparisons presented in Tables 2, 3, in which C_1_ values increased progressively from healthy controls to HBV patients and HCV patients.

## 4 Discussion

### 4.1 Harmonic analysis of radial pulse waveforms in HBV and HCV infections

Chronic HBV and HCV infections are often referred to as “silent” diseases because they may remain asymptomatic for many years before clinically apparent liver dysfunction develops. In the present study, all participants exhibited liver enzyme levels within the normal range, and no significant differences in GOT or GPT were observed among the control, HBV, and HCV groups (Table 1). Nevertheless, significant alterations in radial pulse harmonic characteristics were detected, particularly in C_1_ measured at the Guan position on the left wrist. To the best of our knowledge, this is the first study to investigate harmonic characteristics of RPP signals in individuals with HBV and HCV infections using the Skylark pulse analysis system combined with HA. More importantly, separate subgroup analyses revealed that the HCV subgroup, which primarily drove the significant association observed in the combined viral hepatitis model, whereas the HBV subgroup exhibited a similar directional trend but did not reach statistical significance. These findings suggest that harmonic alterations may reflect subtle physiological changes associated with chronic viral hepatitis, even in individuals without overt liver dysfunction.

From a TCM perspective, the Guan position on the left wrist has long been considered functionally related to the liver system (26). This traditional concept can be traced back to Huangdi Neijing (The Yellow Emperor’s Inner Canon), in which the liver is described as playing a central role in maintaining physiological regulation and bodily function (26). Interestingly, among all six pulse positions examined, only the left Guan position demonstrated a significant group difference in C_1_. Although the physiological basis underlying this observation remains incompletely understood, the findings provide preliminary quantitative evidence supporting the potential relevance of this pulse location in patients with chronic viral hepatitis. Previous studies have demonstrated that HA of radial pulse waveforms may provide clinically relevant information in several chronic diseases. For example, C_0_ has been reported as a protective indicator associated with type 2 diabetes mellitus (22), whereas C_1_ has been associated with coronary artery disease and cardiovascular risk assessment (16). Furthermore, C_1_ has been identified as an independent predictor of major adverse cardiovascular events, cardiovascular mortality, and microvascular complications in previous investigations (13, 19). The fourth harmonic component (C_4_) has also been associated with cardiovascular complications in patients with type 2 diabetes mellitus (17). In addition, HA has been applied to evaluate physiological responses to acupuncture and may detect circulatory alterations earlier than conventional hemodynamic measurements (27).

Taken together, these studies suggest that harmonic components derived from radial pulse waveforms may reflect systemic physiological regulation rather than organ-specific pathology alone. In the present study, C_1_ was independently associated with viral hepatitis status in the combined model and remained significant in the HCV subgroup analysis. While the observed effect size was modest, the findings raise intriguing questions regarding the influence of chronic viral infections on pulse-wave harmonic characteristics, particularly in asymptomatic individuals with preserved liver function. Further studies with larger cohorts and mechanistic investigations are warranted to clarify the physiological pathways linking chronic viral hepatitis and pulse-wave harmonic alterations.

### 4.2 Association between the first harmonic component and viral hepatitis status

Chronic viral hepatitis remains a major public health concern in Taiwan and worldwide. According to the Taiwan Food and Drug Administration (TFDA), hepatocellular carcinoma is one of the leading causes of cancer-related mortality, and chronic HBV and HCV infections are among its most important etiological factors (2). As HBV and HCV infections may remain clinically silent for many years before the development of cirrhosis or hepatocellular carcinoma, considerable effort has been devoted to identifying physiological alterations associated with these infections (28). The identification of C_1_ was based on the sequential findings presented in Tables 2, 3. Among the six pulse positions examined, only the left Guan position demonstrated a significant group difference (Table 2). Among the harmonic components (C_0_–C_10_) derived from this pulse position, only C1 remained significantly different among the study groups (Table 3).

In the present study, exploratory logistic regression analyses identified sex and the first harmonic component as variables independently associated with viral hepatitis status in the combined model. However, subsequent subgroup analyses demonstrated that the observed association was primarily attributable to the HCV subgroup. In contrast, the HBV subgroup exhibited a similar directional trend but did not reach statistical significance. These findings are consistent with the group comparisons presented in Tables 2–4, in which C_1_ values increased progressively from healthy controls to HBV patients and HCV patients.

The observed association between C_1_ and viral hepatitis status should be interpreted cautiously. Although statistically significant, the effect size was modest (OR = 1.008), suggesting that C_1_ alone is unlikely to serve as a stand-alone diagnostic marker. Rather, the findings indicate that harmonic characteristics of radial pulse waveforms may reflect subtle physiological alterations associated with chronic viral hepatitis, particularly HCV infection. Future studies involving larger cohorts and multimodal clinical assessments are required to determine whether these harmonic features provide complementary information beyond conventional clinical and laboratory evaluations. Sex was also independently associated with viral hepatitis status in the combined model. Although the sex distribution did not differ significantly among the three study groups, a higher proportion of male participants was observed in the HBV and HCV cohorts. This observation is consistent with previous epidemiological studies reporting a greater prevalence of HBV-related liver disease and hepatocellular carcinoma in men than in women (29). Nevertheless, given the relatively small sample size and the imbalance in sex distribution, the observed association should be interpreted with caution.

An interesting observation in the present study is that all participants exhibited liver enzyme values within the normal clinical range, and no significant differences in GOT or GPT were observed among the study groups. Despite this, significant alterations in C1 were detected, particularly in patients with HCV infection. This finding suggests that pulse-wave harmonic characteristics may reflect physiological changes that are not captured by routine liver enzyme measurements alone. Further mechanistic studies are needed to clarify the biological basis of these observations.

### 4.3 Clinical implications of the logistic regression findings

Chronic HBV and HCV infections remain major causes of liver-related morbidity and mortality worldwide. In Taiwan, hepatocellular carcinoma is one of the leading causes of cancer-related death, and chronic viral hepatitis is a major contributing factor (2). As HBV and HCV infections may remain clinically silent for prolonged periods before the onset of cirrhosis or hepatocellular carcinoma, there is considerable interest in identifying physiological alterations associated with these infections (28).

In the present study, exploratory logistic regression analyses identified sex and the first harmonic component as variables independently associated with viral hepatitis status in the combined model. However, subsequent subgroup analyses demonstrated that the HCV subgroup primarily drove the observed association, whereas the HBV subgroup exhibited a similar directional trend but did not reach statistical significance. These findings suggest that alterations in C_1_ may be more pronounced in HCV infection, although the relatively small HBV sample size may also have limited statistical power.

The observed effect size of C_1_ was modest (OR = 1.008), indicating that C_1_ alone is unlikely to serve as a stand-alone diagnostic marker. Nevertheless, the persistence of a significant association after adjustment for demographic variables suggests that harmonic characteristics of radial pulse waveforms may capture physiological information not fully reflected by conventional clinical parameters. Notably, age, BMI, GOT, and GPT were not retained in the final multivariable model, despite all participants exhibiting liver enzyme values within the normal clinical range. An additional finding was the association between sex and viral hepatitis status in the combined model. Although no statistically significant difference in sex distribution was observed among the three study groups, a higher proportion of male participants was present in the HBV and HCV cohorts. This observation is consistent with previous epidemiological studies reporting a higher prevalence of HBV-related liver disease and hepatocellular carcinoma among men than among women (29). However, given the relatively small sample size and the imbalance in sex distribution, this finding should be interpreted with caution.

Taken together, the logistic regression analyses support the hypothesis that specific harmonic characteristics of radial pulse waveforms, particularly C_1_, are associated with chronic viral hepatitis. While these findings do not establish diagnostic utility, they suggest that HA may provide complementary physiological information regarding systemic alterations associated with HBV and HCV infections. Larger prospective studies are warranted to validate these observations and clarify their potential clinical significance.

### 4.4 Study limitations

Several limitations of this study should be acknowledged. First, the sample size was relatively modest, particularly after separating participants into HBV and HCV subgroups. The small sample size may have reduced statistical power and increased the possibility of model instability. Consequently, the subgroup analyses should be considered exploratory, and the findings require confirmation in larger independent cohorts. Second, although no statistically significant difference in sex distribution was observed among the study groups, the proportion of male and female participants was not fully balanced. Sex was therefore included as a covariate in the multivariable logistic regression analyses. Nevertheless, residual confounding related to sex cannot be completely excluded. Future studies with larger and more balanced populations are needed to better characterize potential sex-specific effects on pulse-wave harmonic parameters. Third, the present study employed a single pulse diagnosis instrument (the Skylark pulse analysis system), and comparisons with other pulse acquisition systems were not performed. In addition, pulse signal acquisition depends on the accurate positioning of the pressure sensor at the Cun, Guan, and Chi pulse locations. Although all measurements were conducted by trained and experienced personnel following a standardized protocol, minor positioning errors may still have occurred. Furthermore, emotional stress, conversation, and other transient physiological influences may affect pulse-wave characteristics despite efforts to minimize these factors during data collection. Fourth, the pulse-wave measurements in this study were obtained using a fixed-depth acquisition approach designed to capture the most prominent RPP signal. This methodology may not fully replicate the multi-depth assessment traditionally performed in manual pulse diagnosis. Future studies should investigate whether harmonic characteristics vary with pulse depth and whether this information provides additional physiological insights.

Finally, although several demographic and clinical variables were considered in the statistical analyses, comprehensive information regarding medical history, lifestyle factors, and medication use was not available for all participants. Consequently, the potential influence of unmeasured confounding factors cannot be excluded. Future prospective studies incorporating more detailed clinical characterization and external validation cohorts are warranted to confirm the reproducibility and generalizability of the present findings.

## 5 Conclusion

The present study demonstrated that the first harmonic component (C_1_) of radial pressure pulse waveforms was associated with chronic viral hepatitis status, particularly in patients with HCV infection. Significant differences in C_1_ were observed despite normal liver enzyme levels, suggesting that the harmonic characteristics of radial pulse waveforms may reflect physiological alterations associated with chronic viral hepatitis that are not captured by conventional biochemical assessments.

While the observed associations were modest and should be interpreted with caution, the findings provide preliminary evidence supporting the use of HA as a quantitative approach to investigating pulse-wave alterations in HBV and HCV infections. Larger prospective studies are required to validate these observations, explore the underlying physiological mechanisms, and determine their potential clinical relevance.

## Data Availability

The raw data supporting the conclusions of this article will be made available by the authors, without undue reservation.
