# Supplementary material for: Harmonic analysis of radial pulse in traditional Chinese medicine: physiological alterations associated with hepatitis B and hepatitis C infections
Source: Front Med (Lausanne). 2026 Jul 8;13:1848946. doi: 10.3389/fmed.2026.1848946 (PMC13389843; doi:10.3389/fmed.2026.1848946)
Supplement: Supplementary file 1 [file Data_Sheet_1.PDF]

Supplementary material:
Code Sharing

1. We are currently preparing our raw dataset, including radial pressure pulse signals collected from all six measurement sites, for deposition in a public repository such as Figshare or Zenodo. The dataset will include anonymized participant information along with relevant metadata.
2. The custom MATLAB scripts developed for harmonic analysis and peak alignment will also be made available here.

Main Program

```
clear all
hold off
raw=textread('WPP2.txt',' %f');    % Radial Arterial Waveforms input
plot(raw)
[RRI P_rri]=RRI_seq(raw);          % call RRI function; RRI=[t1 t2 t3...t1009]; RR Intervals
                                   %    P_rri=[Pi,P2,P3,...]; P1 the first R wave location;
TN = length(RRI);    % TN is the total number of periods of sampled data
        for j = 1:TN
            n1 = P_rri(j);
            n2 = P_rri(j+1);
            N = n2-n1+1;
            xn = raw(n1:n2);
           % plot(xn)
            hold on
            A0 = mean(xn);
            Xk = dfs(xn,N);          % call discrete Fourier series function
            C(j,:) = Xk(1:11)/A0;
            AmpC(j,:) = abs(C(j,:));
        End
AmpC=mean(AmpC)
```

DFS Subroutine

```
function [Xk] = dfs(xn,N)
% Computes Discrete Fourier Series Coefficients
% ---------------------------------------------
% [Xk] = dfs(xn,N)
% Xk = DFS coeff. array over 0 <= k <= N-1
% xn = One period of periodic signal over 0 <= n <= N-1
% N = Fundamental period of xn
%
n = [0:1:N-1];              % row vector for n
k = [0:1:N-1];              % row vecor for k
WN = exp(-j*2*pi/N);        % Wn factor
nk = n'*k;                  % creates a N by N matrix of nk values
WNnk = WN .^ nk;            % DFS matrix
Xk = xn' * WNnk;            % row vector for DFS coefficientsxn
```
